# Pharmacists’ perceptions of their emerging general practice roles in UK primary care: a qualitative interview study

**DOI:** 10.3399/bjgp17X691733

**Published:** 2017-07-04

**Authors:** Jo Butterworth, Anna Sansom, Laura Sims, Mark Healey, Ellie Kingsland, John Campbell

**Affiliations:** Primary Care Research Group, University of Exeter Medical School, Exeter.; Primary Care Research Group, University of Exeter Medical School, Exeter.; Primary Care Research Group, University of Exeter Medical School, Exeter.; Primary Care Research Group, University of Exeter Medical School, Exeter.; Primary Care Research Group, University of Exeter Medical School, Exeter.; Primary Care Research Group, University of Exeter Medical School, Exeter.

**Keywords:** extended roles, general practice, pharmacists, primary care

## Abstract

**Background:**

UK general practice is experiencing a workload crisis. Pharmacists are the third largest healthcare profession in the UK; however, their skills are a currently underutilised and potentially highly valuable resource for primary health care. This study forms part of the evaluation of an innovative training programme for pharmacists who are interested in extended roles in primary care, advocated by a UK collaborative ‘10-point GP workforce action plan’.

**Aim:**

To explore pharmacists’ perceptions of primary care roles including the potential for greater integration of their profession into general practice.

**Design and setting:**

A qualitative interview study in UK primary care carried out between October 2015 and July 2016.

**Method:**

Pharmacists were purposively sampled by level of experience, geographical location, and type of workplace. Two confidential semi-structured telephone interviews were conducted — one before and one after the training programme. A constant comparative, inductive approach to thematic analysis was used.

**Results:**

Sixteen participants were interviewed. The themes related to: initial expectations of the general practice role, varying by participants’ experience of primary care; the influence of the training course with respect to managing uncertainty, critical appraisal skills, and confidence for the role; and predictions for the future of this role.

**Conclusion:**

There is enthusiasm and willingness among pharmacists for new, extended roles in primary care, which could effectively relieve GP workload pressures. A definition of the role, with examples of the knowledge, skills, and attributes required, should be made available to pharmacists, primary care teams, and the public. Training should include clinical skills teaching, set in context through exposure to general practice, and delivered motivationally by primary care practitioners.

## INTRODUCTION

UK general practice is experiencing a workload crisis. Over half of GPs aged >50 years intend to leave the profession within 5 years,^1^ and GP training applications fell by 15% between 2013 and 2014.^2^ Although pharmacists are the third largest healthcare profession in the UK,^3^ their skills are being underutilised.^4^ This interview study forms part of a project that aimed to investigate the potential of integrating pharmacists into general practice, to relieve workforce pressures as advocated by a UK collaborative ‘10-point GP workforce action plan’.^5^ Table 1 illustrates the historical context of pharmacists’ evolving roles in UK general practice.^5^^–^7 Most recently, the Royal College of General Practitioners reported that strong progress is being made with an NHS England pledge to invest an additional £122 million in the current pilot scheme for practice-based pharmacists, in order to deliver a further 1500 pharmacists by 2020.^8^

**Table 1.** Timeline of the developing role of pharmacists in UK primary care

**When**	**Report/policy released^5^^–^8**	**Outcomes generated**
**1986**	The Nuffield Report highlights dramatic underutilisation of UK pharmacists^6^	Suggestions that collaboration between GPs and pharmacists could improve effectiveness and reduce costs of prescribing
**1990s**	NHS health reforms result in the allocation of drug budgets to individual health authorities	Interest from general practice to use clinical pharmacists within primary care
**2005**	NHS Community Pharmacy contract	Increases the range of services community pharmacists can provide
**2008**	The pharmacy workforce census	Seven per cent of pharmacists are already working within general practices or for local health authorities
**2015**	NHS England, the General Practitioners Committee of the BMA, Health Education England, and the Royal College of General Practitioners reveal their *Ten Point GP Workforce Action Plan* 8	The Clinical Pharmacists in General Practice Pilot aims to utilise pharmacists’ knowledge and expertise surrounding medications, in order to complement the knowledge and roles of GPs and practice nurses in general practice surgeries
**2017**	RCGP *GP Forward View* Interim Report 8	NHS England reports that >85% of pharmacy professionals have received training in the use of the patient Summary Care Record, and that all pharmacists will have received training by March 2017
**By 2040**	The Centre for Workforce Intelligence	Estimates that oversupply of pharmacists could reach 11 000–19 000 due to increased numbers of pharmacy students over the last decade

**Figure 1.**
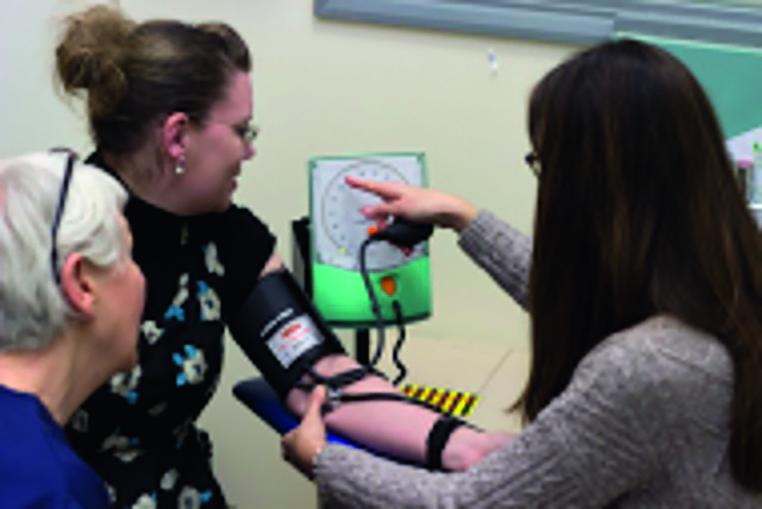
***Pharmacists in general practice roles.***

UK literature exploring pharmacists working in primary care is sparse, due to the relatively new and evolving nature of the role. In 2014 a systematic review of international studies revealed that pharmacists co-located in general practice clinics could deliver favourable results when conducting chronic disease clinics and quality use of medicines reviews, resulting in improved clinical outcomes such as blood pressure, blood sugar, and cholesterol reduction (Figure 1).^9^

A training programme for pharmacists interested in extended roles in primary care was developed through an NHS Education South West-funded project carried out by the University of Exeter Medical School. It was delivered face to face and consisted of 6 full days completed over 6 consecutive months, with associated self-directed learning. A GP and a pharmacist were the main course facilitators, and practice nurses were also involved in delivery of course content. Taught elements encompassed practical clinical skills training alongside theoretical teaching on acute illness, long-term conditions and multimorbidity, evidence-based practice, pharmacology in primary care, the organisation, structure, and delivery of general practice, and the importance of patient experience. There was also an opportunity to attend a 1-day placement in general practice. Appendix 1 outlines the course programme. The training programme was evaluated through a mixed-methods approach using study day feedback, multiple-choice question assessment, and qualitative interviews with participants.

Changes in the skill mix and delegation of duties between primary care practitioners has previously been seen in the UK, notably during the introduction of nurse practitioners.^10^ There have, however, been few studies that have specifically investigated the integration of pharmacists into general practice roles. This qualitative study aims to explore pharmacists’ perceptions of primary care roles, including the potential for greater integration of their profession into general practice and the future potential of this role.

**How this fits in**

This study used qualitative methods to contribute to the currently sparse literature on pharmacists’ perceptions regarding the integration of their profession into UK general practice. The study shows that there is enthusiasm and willingness, especially among junior pharmacists, for new, extended roles based in primary care, which should be harnessed in order to relieve current GP workforce pressures. A definition of the role, with examples of the knowledge, skills, and attributes required, should be made available to pharmacists, primary care teams, and the public. Primary care training for pharmacists should include clinical skills teaching, set in context through exposure to general practice, and delivered motivationally by primary care practitioners.

## METHOD

The study took place between October 2015 and July 2016. A database of pharmacy contacts in the South West (Devon, Cornwall, and Somerset) was created using local pharmaceutical committee and clinical commissioning group emailing lists, as well as social media, in order to inform pharmacists about the training programme. Written applications were used to collect information from interested pharmacists, including current role, qualifications, and future career plans. This enabled purposive sampling by level of experience, geographical location, and type of workplace. Three researchers independently scored the applications by level of motivation, experience, and career plans. There were 16 course places available. Those applicants with the highest scores were selected to attend the course and were invited to participate in two confidential telephone interviews, one before and one after the course.

Written informed consent was obtained prior to undertaking interviews. A semi-structured interview guide was used (available from the authors on request) and interviews were digitally audio-recorded and transcribed verbatim. Interviews were listened to repeatedly in order to improve transcription validity.

A constant comparative, inductive approach to thematic analysis was used.^11^^,^^12^ NVivo (version 11) was used to organise the data. New fragments of coding were constantly compared with old data in order to construct new common themes,^12^ and to make cautious propositional statements. No new themes emerged during analysis of the final transcripts, thus theoretical saturation was considered achieved.

## RESULTS

Of the 1050 pharmacists contacted by e-mail, 38 replied and 16 were purposively selected for the training programme. All 16 agreed to participate in interviews but one could not complete the post-course interview due to personal circumstances. Table 2 shows participants’ characteristics. Many participants had little or no prior experience of working in a general practice surgery.

**Table 2.** Participants’ characteristics, *n* = 16

**Variable**	**Number**
**Sex**	
Male	5
Female	11

**Age**	
21–30	3
31–40	4
41–50	6
51–60	2
Not given	1

**Job role on course application^a^**	
Community pharmacist	7
CCG pharmacist working in general practice	5
CCG pharmacist not directly working in general practice	4

**Postgraduate awards held**	
Clinical diploma	5
Independent prescribing certificate	6
Other	6

a *Community pharmacist role included dispensing; over-the-counter minor ailments advice; advanced services such as medicines use reviews, new medicine service, emergency hormonal contraception, and chlamydia screening; general practice role included conducting minor illness and long-term condition clinics, telephone consultations, liaising with community and hospital colleagues, and pain medicines optimisation clinics; CCG role included prescribing management planning, practice audits, assessing practice compliance to formularies and guidelines, and reconciling prescribing problems with GPs. CCG* = *clinical commissioning group.*

Pre-course and post-course interviews lasted 30 to 45 minutes. There were no significant problems with conducting interviews or with the quality of transcription data.

The main themes that emerged from the data related to participants’ initial expectations of their role in UK general practice, the influence of the training course on their perceptions, and their predictions for the future of this role.

### Expectations of the role

Before the training programme, participants varied regarding what they defined the role of a pharmacist working in primary care to be. Definitions included: practice-based pharmacists conducting patient clinics and home visits; community pharmacists providing written advice to GPs; and clinical commissioning group-employed pharmacists running prescribing checks on practice databases. Many saw the role as a means to reduce GP workload and to enable more efficient and effective patient care.

Participants recognised the importance of being part of a multidisciplinary team but drew clear distinctions between themselves and other practitioners:

‘That’s very much what I see my role as; that I’m here to look after the GPs so that they can actually provide the function. Yes I do my own things — I run clinics, I see patients — but ultimately I’m here to facilitate them being able to do their role better. By doing that the whole service is improved.’

‘We are not little doctors, we are a completely different animal … Ultimately in the same way as a nurse is still a nurse, an OT is still an OT, we are still pharmacists … we serve very different functions.’

‘The doctor had a view of the diagnosis and the nurse was very holistic in her approach towards the patient, I was very focused on the drugs, and the dietician had her perspective as well … so yes there were overlaps but our functions and our roles, erm, I think we’re better when we work together. I don’t consider us to be in competition.’

(Ph12)

Many felt they were in the best position to address medication adherence, through applying their knowledge of packaging options or frequency of administration, to reduce polypharmacy to safer, more manageable numbers of prescribed medications per patient, and to monitor prescribed medications that could otherwise lead to adverse events. Some felt that pharmacist prescribers were more likely than other practitioner prescribers to meet government cost-effective prescribing agendas, by maintaining their knowledge of drug formularies and medicines availability.

Most participants reported a limit to the level of their proposed responsibilities: being willing to take a proactive approach to medicines optimisation, using biochemistry results and therapeutic drug monitoring, but wanting to pass complex diagnostic decisions to a GP. Some discussed potential difficulties attaining clinical management decisions, however, particularly if addressing a whole team of GP partners during a clinical meeting. The importance of maintaining good working relationships and written communication skills was reflected upon.

Participants recognised that there were current gaps in their knowledge, skills, and attributes required to perform an extended role in general practice, however, those not yet working in primary care could not always identify specific learning needs:

‘Within community pharmacy, my observation is that the pharmacists there aren’t really familiar with their limitations. They feel that they’ve got a bigger role because they’ve had such an extensive training but they don’t realise the gaps in their knowledge, don’t know what they don’t know … and most pharmacists actually don’t know what goes on in a GP surgery. They don’t know what the actual function of the GP is.’

(Ph2)

Participants varied in levels of expressed self-confidence for working in general practice. Greater confidence appeared to be associated with greater experience and qualification, and with a willingness to accept more responsibility in respect of patient care.

When asked about perceived barriers to working in primary care, community pharmacists expressed concerns that patients might perceive pharmacists as ‘over-the-counter medicines dispensers’ and might not trust their clinical decision making in general practice. Some primary care pharmacists discussed examples of having their opinion rejected by a GP. Participants felt that positive promotion of the role among patients and other primary care practitioners was still needed. However, it was commonly perceived that pharmacists working in primary care have high levels of job satisfaction associated with increased patient contact and holistic practice:

‘We’ll be working with GPs and it’s quite nice and encouraging to hear it from a GP’s mouth … To hear that “actually we want you, we need you and there’s room for you guys. That it’s not going to be that we’re threatened that you’re going to take over our jobs and take our money” … I think in the past that was the fear, especially with the whole initiative for pharmacists to start prescribing. A lot of GPs were very anti that … the GPs’ attitudes have changed enormously.’

(Ph6)

### Influences of the training course

Several participants’ perceptions of a pharmacist’s role in general practice were influenced by their participation in the training programme described previously.

After completing the course, most participants felt that a formal, standardised qualification in primary care pharmacy would improve their employability by practices and their access to indemnity cover. Many expressed enjoyment in continuing professional development but described a lack of available courses in primary care:

‘They would like something that says I am competent to deal with these five minor ailments, I have done a 6-day training programme specifically on eyes, ears, chests, throats, noses, or whatever it is. I have looked at 16 case scenarios, I’ve had an 80% success in my multiple choice question paper that says that of all these issues that I can come across, I knew them, and I now feel competent.’

(Ph10)

Several participants acknowledged a characteristic ‘risk-averse’ approach that they applied to their work. The course raised their awareness of a need to manage uncertainty in clinical care and to use critical appraisal, within the bounds of their clinical competence, to flexibly manage situations that could not be approached according to guidelines. Some reported that the course had given them the confidence to do this, because of the motivational delivery of its content by a GP facilitator and because of the clinical skills teaching, reported to be otherwise unavailable in the context of primary care:

‘The level of responsibility and accountability is different to what we’ve been used to. You’re really looking at the same coin from entirely the other side because we are used to constantly checking what other people are doing and suddenly you’d be the person doing the doing, as it were — it’s a very different role.’

(Ph9)

Less experienced participants valued the opportunity to network with those pharmacists who were already working in primary care, enabling them to put the training into context and to visualise the potential of their future role. However, those participants already working in primary care reported that the course had little impact on their attitudes towards their current role and that it had not changed the way that they practise or their outlook for the future:

‘I guess it kind of encouraged me not to be afraid to dream big because anything is possible in this age we’re in. But I would definitely like to progress in primary care.’

(Ph6)

### Predictions for the future

Participants’ predictions for the future emerged as a common theme across both pre- and post-course interviews. Participants recognised a need to be adaptable with regard to the uncertainty of their future roles. They viewed this as a challenge for a professional group who are used to following clear structures and processes in their careers. They felt that GP practices were likely to have individualised views on the tasks expected of a pharmacist in their team:

‘I think if the government, the NHS, are serious about trying to manage the resources that they’ve got, then there have to be changed roles for people; people coming out of their boxes and breaking down the barriers.’

(Ph16)

Many participants anticipated an increase in the numbers of pharmacists with independent prescribing skills. Others visualised practice pharmacists becoming gatekeepers: liaising with community pharmacists and addressing medication queries using their access to patient notes. Several speculated about allocating more time per patient encounter than is currently possible for a busy GP and managing their own appointments to ensure better continuity of care. However, those with experience of general practice acknowledged logistical factors that limited these ideal circumstances including shortages of clinic rooms and high levels of patient demand.

Participants had high expectations of their future roles with regard to patient and practitioner outcomes including improvements in job satisfaction, patient health, patient satisfaction with health care, patient enablement for self-management, and reductions in costs to the NHS:

‘I think there are a lot of people who will need to be brought over to our side to realise what our potential is; what we are capable of doing; what we are experts in; and what we can really offer to improve outcomes.’

(Ph35)

A common perception was that the future of a pharmacist’s primary care role was to fulfil public health agendas, to ensure government standards regarding health checks, for example, and to reduce polypharmacy, particularly in respect of vulnerable groups such as older patients and those with multimorbidity.

Many participants reported that they would value the opportunity for team working, reflecting on the relative isolation from clinical care experienced in community pharmacy. Some felt that working as the only pharmacist in a practice might bring a different type of isolation, however. Some wanted to become leaders in their field, to inspire others to join them in a general practice environment.

The subject of salary was discussed. The pay ‘banding’ currently advertised in many primary care posts would mean a drop in salary for an experienced community pharmacist but was on par with hospital pharmacy. Pharmacists employed by the clinical commissioning group often had different job descriptions and salaries from those employed privately by GP practices. Pharmacists perceived ongoing differences in the ‘cost-effective versus clinically effective’ priority weightings of their workload by different employers.

Participants had varied perspectives on how they saw their personal careers unfolding in primary care. Overall, it appeared that community pharmacists, and those just beginning new roles in primary care, were open to acquiring new skills in order to extend their roles into minor ailments, triage, and clinical examination, for example. However, those who had been established in primary care roles for some time were much less willing to extend their skills beyond medicines management and medicines optimisation tasks:

‘I feel quite strongly that we shouldn’t be trying to develop skills that we don’t have in terms of, in areas that we are not experienced. So medicines are our training, that is what we know, so we should be trying to do everything we can to make sure medicines are prescribed safely and appropriately, not trying to diagnose musculoskeletal pain.’

(Ph38)

All participants agreed that a clear vision for the future was required nationally and that it needed to be communicated widely among their professional group.

## DISCUSSION

### Summary

Participants’ perceptions varied by degree of experience of working in primary care, and by current working role. Therefore there was wide variability in their understanding of the role of the pharmacist in UK primary care and in the way that the training course influenced their perceptions. All participants recognised challenges and uncertainties regarding the future direction of the role, and their degree of experience, along with the training they received, influenced their confidence for this type of work. There were also several perspectives regarding the skills, knowledge, and attitudes required for a role in primary care, as well as the potential outcomes that might be achieved. However, all participants appeared willing to contribute to the relief of workload pressures on GPs and on the primary care team.

The most recently qualified pharmacists, and those with less experience of working in primary care, appeared the most willing to engage with training and with career opportunities for extended roles in general practice.

### Strengths and limitations

This study used qualitative methods to contribute to the currently sparse literature on pharmacists’ perceptions regarding the integration of their profession into general practice in the UK.

The sample size for interviews was predetermined by the number of available places on the training course. However, the sample size is comparable to previous studies using similar methodologies,^13^ and was sufficient to have achieved saturation.^14^ There was heterogeneity by age, type of employment, and previous level of qualification. Participants had a high degree of interest in the subject area, which provided rich material for qualitative analysis. The schedule for development of the training programme, marketing, and commencement of the course was tight. With a longer recruitment period there may have been a greater uptake for the course. However, the authors believe that there were sufficient numbers and sufficient heterogeneity of participants, with high scores on their application forms, to fill the 16 available places at the time of commencement.

It was apparent that participants had different perceptions regarding what constitutes ‘primary care’ during early interviews, and the interviewer subsequently sought to clarify the context with participants.

The possibility of findings being geographically specific, with participants recruited from Devon and Cornwall, was considered. However, many of the pharmacists discussed training or working in other locations around the UK. Heterogeneity of the sample (by age, level of qualification, and previous general practice experience) allows the applicability of emergent themes to be considered in a wider context.^15^

Because of their workload, different researchers conducted the pre-course interviews and post-course interviews. Participants were not informed of this unless they asked and this occurred on one occasion at the end of the postcourse interview. Positional reflexivity was demonstrated through reflective notes and critical discussion between authors.^16^^,^^17^ A GP researcher was able to interpret interview content from a clinical perspective, which was useful during analysis.

### Comparison with existing literature

The participants expressed uncertainty regarding the definition of a pharmacist’s role in primary care. The UK literature is currently sparse because of the evolving nature of the role, therefore, with acknowledgement of the differences in pharmacists’ primary care roles internationally,^9^ comparisons were drawn with the international literature. In Canada, Jorgenson *et al* 18 used qualitative methods to identify that a pharmacist’s lack of clarity about, or knowledge of their primary care role, negatively affected their potential integration into the primary care team. They also highlighted that difficulties with patients’ access to the pharmacist, influenced by resources and funding, could also have a negative impact.^18^ The more experienced participants in the current study also reported the same difficulties.

Several participants who had little or no experience in primary care were concerned about how they might be perceived in this new role by other members of the primary care team. Jorgenson *et al*^18^ identified that a pre-existing relationship between the pharmacist and the general practice in which they will work, and the primary care team’s perceptions of the pharmacist’s confidence, assertion, and visibility in the team, could have a positive influence on their successful integration. Conversely, perceived resistance by doctors to accept or to trust the pharmacist in their new role, and a lack of managerial support, had a negative impact. An Australian study additionally emphasised that having time to build trust in a pharmacist was of importance from the perspective of both practice staff and patients.^19^ The participants in the current study also felt that continuity with patients and maintaining frequent communication with other members of the primary care team were important. The Canadian IMPACT project^20^ emphasised the importance of pharmacists spending time in general practices during their training, particularly highlighting the relevance to the development of pharmacists’ identity in primary care settings.

The UK’s Centre for Pharmacy Postgraduate Education has a ‘Declaration of Competence’ system developed to support pharmacists in assuring commissioners that they have the appropriate knowledge, skills, and behaviours to deliver high-quality, consistent services.^21^ Several core competencies for a pharmacist working in primary care have also been outlined by a Canadian Delphi study, relating to communication, collaboration, and professionalism, with an emphasis on direct patient care.^22^ The participants in this study discussed the skills, knowledge, and attitudes that they had gained from the course in respect of these competencies, as well as reflecting on the relevance of the training when undertaking patient care in a general practice surgery.

Some of the participants, particularly those who were more experienced, or who were already working in primary care, felt that their role should focus on their expertise in medicines management and medicines optimisation. Consultations with a pharmacist regarding medications, in a general practice setting in the UK, have previously been reported to be rich in content, acceptable to patients, and perceived by pharmacists to be a possible way to extend their role.^23^ The first randomised controlled trial of pharmacist prescribing in the UK suggested that there may be a benefit for patients with chronic pain.^24^ A UK analysis of audio-recorded consultations about medications, between patients and pharmacists in general practice, concluded that pharmacists were patient centred, and responded positively and effectively to patients’ emotional cues and concerns.^19^ The participants in the current study recognised the importance of a holistic, individualised approach to patient care and they valued the communication skills training on this course.

The participants were very aware of safe practice, and were seeking to ensure that they were working within the boundaries of their own clinical competence. They discussed the implications of extended roles on their indemnity cover as well as in the context of salary. The PINCER study,^25^ evaluating a pharmacist-led, information technology-based intervention, showed that this approach can reduce medication errors in general practice, when targeting patients on high-risk medications. Similarly, a cluster randomised trial of a pharmacist-led intervention for patients with established heart disease showed improved prescribing of statins and improvements in target cholesterol levels.^26^ Indemnity companies are working to finalise fees relating to the specific roles that a primary care pharmacist might undertake. GP practices vary in the salaries paid to current primary care pharmacists.

### Implications for research and practice

There is enthusiasm and willingness, especially among junior pharmacists, for new, extended roles based in primary care. Promotion of the role among pharmacists, primary care teams, patients, and commissioners is likely to encourage training in general practice roles. This in turn could help to reduce uncertainty regarding definitions of the role, and to fuel pharmacists’ primary care-based career aspirations. A working definition of the role, with clear examples of the knowledge, skills, and attitudes required, should be made readily available to pharmacists, primary care teams, and the general public. This would enable standardised payment bandings and indemnity fees to be developed and applied nationally across primary care. An interim assessment for NHS England’s ‘GP Forward View’ suggests that ‘the success of many of the pledges made … will depend on frontline GPs being aware of how to access certain programmes … for example the roll-out of practice-based pharmacists’.^8^

From the findings of this study, the authors suggest that future training programmes should seek to target pharmacists who are soon to be commencing new roles in general practice surgeries. The use of experienced primary care pharmacists as course facilitators would be well received by trainees. Courses should include clinical skills training, including those skills applicable to the management of minor ailments and to chronic disease clinics; spending time shadowing and practising skills in a general practice environment; receiving teaching from other primary care practitioners; and a discussion of the attitudes and professional values required for the role. The course should be delivered with a motivational approach in order to positively influence pharmacists’ hopes and expectations for the future of the role, and should be set in context through the use of clinical case scenarios. Pharmacists should also be encouraged to complete a prescribing certificate.

In addition, further research is needed to assess the impact on GP workload of effectively training pharmacists to work in primary care roles in general practices. Further qualitative work could usefully assess pharmacists’ experiences of commencing a new role in general practice, and compare the experiences of those who have or have not recently completed a primary care training programme such as this.

The findings from this study have the potential to inform the successful integration of pharmacists into primary care roles, working in general practices in the UK, in order to help relieve the current workload pressures on GPs.

## Appendix 1. Training course programme for pharmacists interested in extended roles in primary care

**Table:**

**Day 1** Valuing the primary care pharmacist: The skills and role of the primary care pharmacist in a primary care team Leading on prescribing quality improvement changes Managing the patient with respiratory infections Managing the patient with eye symptoms
**Day 2** Clinical skills training 1: ENT and asthma/COPD annual reviews
**Day 3** Principles of chronic disease management Hypertension annual reviews Cardiovascular risk and lifestyle modification: Using CVS risk profiling in practice and preventive prescribing Communication skills for encouraging lifestyle changes
**Day 4** Medicines management and optimisation in practice: Medicines management and patient enquiries Quality improvement of prescribing practice and systems Applying optimisation principles in polypharmacy and multimorbidity Assessments to aid medication reviews and de-prescribing
**Day 5** The diabetic annual review Common dermatological conditions High-risk drugs, rheumatoid arthritis, osteoporosis, and blood test interpretation: Rheumatoid arthritis annual reviews and osteoporosis medication High-risk drug reviews, audits and interpreting blood results
**Day 6** Clinical skills and communication training 2: successful integration of pharmacists in primary care roles/local networks and continuing professional development

COPD = chronic obstructive pulmonary disease. CVS = cardiovascular. ENT = ear, nose, and throat.
